# Arthrodesis of the Proximal Interphalangeal Joint of the Finger—A Biomechanical Study of Primary Stability

**DOI:** 10.3390/jpm13030465

**Published:** 2023-03-02

**Authors:** Michael Millrose, Hans Christoph Vonderlind, Andreas Thannheimer, Till Ittermann, Johannes Rüther, Maximilian Willauschus, Hermann-Josef Bail, Andreas Eisenschenk

**Affiliations:** 1 Department of Trauma Surgery and Sports Medicine, Garmisch-Partenkirchen Medical Centre, 82467 Garmisch-Partenkirchen, Germany; 2 Department of Orthopedics and Traumatology, Paracelsus Medical University, 90471 Nuremberg, Germany; 3 Department of Trauma Surgery, HELIOS Hospital Schwerin—University Campus of MSH Medical School Hamburg, 19055 Schwerin, Germany; 4 Institute for Community Medicine, SHIP/Clinical-Epidemiological Research, University of Greifswald, 17475 Greifswald, Germany; 5 Department of Hand Surgery and Microsurgery, University Medicine Greifswald, 17475 Greifswald, Germany

**Keywords:** arthrodesis, proximal interphalangeal joint, primary stability arthrodesis, biomechanical analysis

## Abstract

Background: Osteoarthritis of the proximal interphalangeal (PIP) joint of the finger often leads to global hand-function detriment. Different techniques for the arthrodesis of the proximal interphalangeal joint have been described that all lead to union in a reasonable percentage of patients and period of time. This biomechanical study aims to analyze and compare the primary stability of different techniques of arthrodesis to render postoperative immobilization unnecessary. Methods: Arthrodeses of 40° of composite cylinders were tested with different techniques in four-point bending for stability in extension as well as flexion. Results: In extension, the compression screw and the compression wires showed the highest stability—whereas in flexion, plate fusion was superior. Tension band, cerclage or compression screw fusion showed the best compromise in flexion/extension stability. Conclusions: Fusion techniques that apply compression to the fusion show superior stability. Cerclage, tension band and compression screws might be able to provide enough stability to withstand the forces exerted during unencumbered activities of daily living. Arthrodesis with plates should be limited to patients with special indications and require immobilization during consolidation.

## 1. Introduction

Osteoarthritis of the proximal interphalangeal (PIP) joint of the finger—either primary or secondary—can cause pain with a concomitant limited range of motion, which often leads to global hand-function deterioration [1,2]. Secondary osteoarthritis is most frequently caused by post-traumatic changes, followed by chronic instability inflammatory diseases [3]. Treatment options, beside conservative treatments such as physiotherapy and non-steroidal anti-inflammatory drugs (NSAIDs), include joint-preserving surgeries such as denervation or different arthroplasties [4]. Joint replacement options include prosthesis or arthrodesis [5]. In particular, the loss of bone stock after failed prostheses leads to the need for arthrodesis [6]. In case of a need to grip things forcefully—as, for example, for artisans—arthrodesis presents a good treatment option. The aim of all these treatments is pain reduction, leading to improved global hand function.

During recent years, research into the PIP joint arthrodesis of the fingers has been characterized by the search for improved implants and techniques with improved stability, combined with simple—as well as tissue-sparing—surgical techniques. Various surgical procedures are currently available for arthrodesis of the proximal interphalangeal joint (PIJ); these procedures, which can achieve good functional results when indicated and used correctly, have their respective advantages and disadvantages. There are techniques which apply additional compression on the arthrodesis gap, such as tension bands or compression screws of different designs [7,8]. In particular, in trauma cases with a destroyed PIJ such as transarticular amputations, implants which can be applied quickly and induce less additional soft tissue damage are chosen. Those implants, typically k wires, do not apply any compression [9]. Plate arthrodesis is of particular importance in the case of segmental bony defects, non-unions and/or after failed endoprosthesis treatments as it can be easily combined with bone grafts, which in turn enable efficient length maintenance, and thus, less shortening of the finger [10]. A recent systematic review on the PIP joint arthrodesis showed that all these procedures lead to union in a reasonable percentage of patients and periods of time [11]. Newer published techniques, which all rely on the principles of established techniques, have not been able to prove themselves superior [8,12]. Due to the wide variety of surgical techniques for the arthrodesis of this joint, we can state that an optimal procedure has not yet been introduced.

Since almost all current techniques require immobilization after surgery of different time periods until bony fusion is reached, there are concerns that adjacent joints might suffer in mobility [13,14]. The aim of most techniques in hand surgery is the early functional treatment and normal usage of the respective hand. Previous studies have shown that most daily activities create forces of up to 20 N [15,16]. If the primary stability of the implant provided is given, one should be able to allow early functional treatment.

Therefore, this biomechanical study aims to analyze and compare the primary stability of different techniques of arthrodesis for the PIP joint of the finger to recommend techniques which qualify for further clinical studies in order to render a postoperative immobilization unnecessary.

## 2. Materials and Methods

The specimens were made from 42 mm-long phalangeal equivalents from short fiber-reinforced epoxy cylinders with a 10 mm outer diameter and a 1.5 mm wall in the fourth generation (Sawbone Europe AB, Malmoe, Sweden). The composite cylinders were filled with cellular rigid polyurethane-foam filling (strength 15) to mimic cancellous bone.

Each arthrodesis was performed at a standardized angle of 40°—a compromise between the recommended arthrodesis angles of the PIJ from 20–50°, depending on the finger [17]. The angle was set by cutting the distal end of each cylinder to 20°.

The tested arthrodesis techniques’ respective implants were crossed K wires, crossed compression wires, intraosseous wiring, tension band wiring, compression screws and anatomical fixation, as well as locking grid plates.

The used K wires were medical stainless steel and 1.2 mm in diameter. The compression wire used had a threadless section 10 mm in length (Koenigsee, Allendorf, Germany). The arthrodesis was achieved with three turns of the trailing thread inserted into the cortical bone to ensure a standardized compression. Both wire arthrodesis techniques crossed the joint at an angle of 45° in the radio-ulnar plane; they were drilled 8 mm proximal or distal, respectively, to the arthrodesis site.

The compression screw (2.2 mm diameter, 26 mm length; Medartis, Basel, Switzerland) was inserted proximal-to-distal, perpendicular to the arthrodesis site to achieve compression. It was angulated to be fully buried into the cylinders. It consisted of two threads of a different pitch and a threadless section of 10 mm, which provided a secure and rigid cortical fixation, as well as compression of the arthrodesis. The diameter varied from 1.0 mm at the tip to 1.8 mm at the second thread. The placement of the compression wire was possible with the 50 mm-long positioning tip. The threads and main part of the compression wire followed the tip until compression was applied onto the fusion site.

Intraosseous wiring and tension band wiring were carried out with a cerclage wire of 0.8 mm and a K-wire of 1.2 mm according to well-described techniques [18,19].

Plate fixation, anatomical as well as locking grid, used a 0.8 mm low-profile plate (grid fixation plate 4 × 2), bent to accommodate the cylinder and the desired angle of 40°. To achieve fixation on both sides of the arthrodesis, 8 cortices were drilled and fixed by 1.5 mm cortical-respective locking screws (Medartis, Basel, Switzerland).

**Figure 1:**
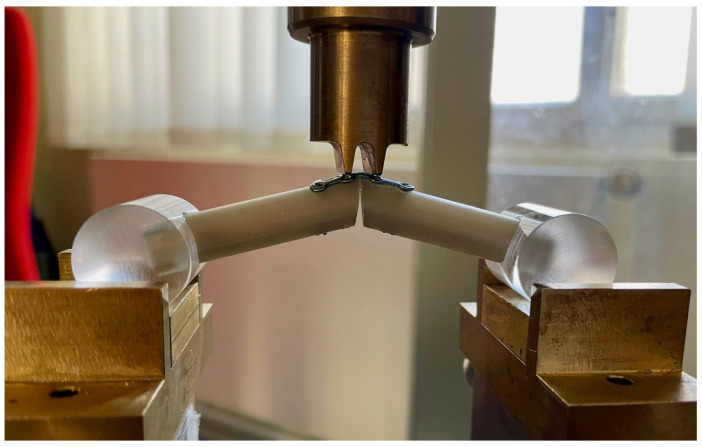
Four-point bending of a composite cylinder arthrodesis in 40° fused with a locking plate.

Using a universal testing machine (Zwick Z050^®^, Zwick GmbH & Co. KG, Ulm, Germany), tests were run in four-point bending. The extensions of the force conductor were positioned 3 mm proximally and distally from the arthrodesis site on the composite cylinders. The ends of the proximal and the distal specimen were inserted into acrylic cylinders (Figure 1). A cylinder was chosen to achieve compensatory rotational movement and a constant contact area during loading. After both composite cylinders were fused with one of the above-mentioned techniques, force was applied continuously at a rate of 100 mm/min in steps of 0.5° until a maximum of 10° flexion or extension was achieved, and the required force was recorded by a computer using testXpert III 1.1 software (Zwick GmbH & Co. KG, Ulm, Germany).

The analyzed data was the force necessary to achieve a bending of 10° either in flexion or extension. For each angle and technique, the mean extension and flexion forces over ten measurements in 0.5° steps were reported. For the angles 37.5°, 35.0°, 32.5° and 30°, differences in mean extension levels between the K wire and all the other techniques were tested using paired t-tests. For mean flexion levels, differences between the techniques for the angles 42.5°, 45.0°, 47.5° and 50° were tested. *p* < 0.05 was considered statistically significant. Furthermore, mean extension and flexion levels were plotted against all measured angles for each technique using a lowess smoother. The statistical analysis was performed using STATA 17.0 (Stata Corporation, College Station, TX, USA).

## 3. Results

Each technique was tested in 10° flexion or 10° extension with *n* = 10, respectively. The strength needed to bend the arthrodesis to the desired angle was measured in 0.5° steps (Table 1 and Table 2).

In extension, the crossed compression wires as well as the compression screw provided the best primary stability, with a bending force necessary to achieve 10° of extension in a 40° arthrodesis of over 68 N. Both are techniques which apply compression to the fusion site. Both plates show the lowest primary stability, at 5.3 N for the fixation plate and 14.69 N for the locking plate, respectively.

In flexion, the data show that both plates provided by far the highest resistance to additional bending, at 118 N each. Both techniques using wires for fusion showed the lowest primary stability, at 24.4 N for the normal k wires and 29.6 N for the compression wires.

**Figure 2:**
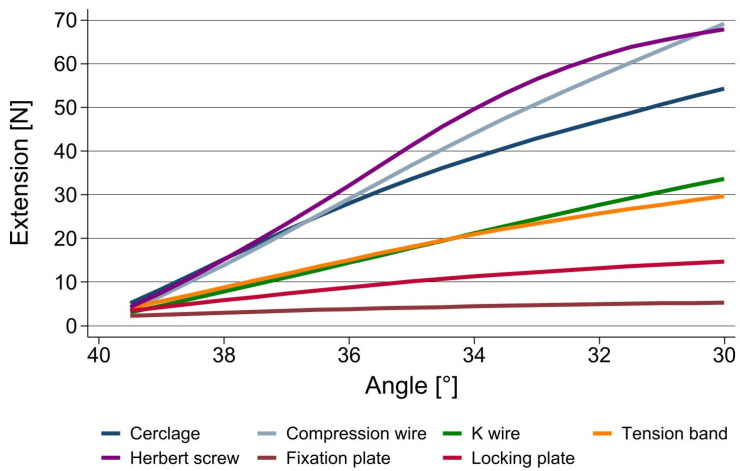
Stability of different fusion techniques for PIP joint arthrodeses in extension.

**Figure 3:**
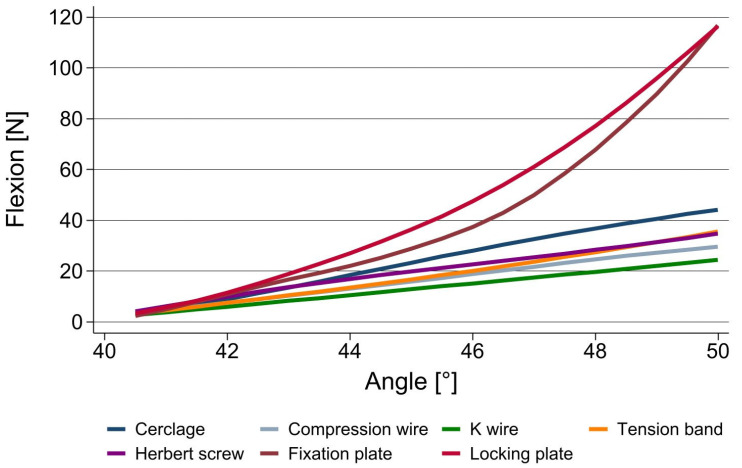
Stability of different fusion techniques for PIP joint arthrodeses in flexion.

The cerclage as well as tension band technique and the compression screw provided good results in terms of their primary stability in extension as well as in flexion (Figure 2 and Figure 3).

The results of the statistical analysis are shown in Table 3 for extension and Table 4 for flexion. K wire fusion was the standard technique chosen to compare against, as it is the best documented technique in the literature and can be used in all situations necessitating arthrodesis.

It could be shown that only the compression screw was statistically significantly more stable in both bending directions than the standard technique. The superiority of the compression wire and the cerclage in extension could also be proven.

## 4. Discussion

This biomechanical study of the primary stability of the proximal interphalangeal joint of the finger with different fusion techniques showed that all the used techniques beside the plates could withstand the forces which are applied during activities of daily living. The implants tested in this study—which are all standard techniques for different indications of joint fusion—demonstrated increasing resistance to bending stress, which can be at least partially attributed to the implant itself.

Although all the used implants have proven themselves to be capable of fusing the PIP joint of the finger in several clinical studies—as shown in the systematic review of Millrose et al.—some are used in special circumstances [12]. For example, plates—regardless of whether they are of a fixation or locking design—show a tendency to lead to tendon adhesions—especially when applied near or on a joint, which might impair the function of the hand [20]. In the special indication of a joint fusion which requires a corticocancellous bone transplant in order to retain the length of the finger, such after a failed prosthesis, a plate can prove itself useful, as it is able to provide a stable fixation of the joint as well as of the transplant [21]. In our study, we were able to show that fusions with plates should be immobilized for the entire consolidation time, as they are not able to adequately withstand extension forces; this is probably because of the low profile of the used plates (0.8 mm) and the fact that the implant crosses the arthrodesis site. However, one must keep in mind that in the phalanges you should not use any stronger implants, due to the above-mentioned complication.

As has been shown in biomechanical analyses of fracture fixation in the phalanges, normally, K wires are able to provide enough stability to allow for early active movement [22]. In our study, we were able to show that they could provide enough stability in extension but lack primary stability in flexion for PIP joint fusions; as such, these should also—at least in the beginning—be immobilized.

Leibovic et al., stated after a clinical study in 1994, that techniques with compression of the fusion site could provide more reliable results [23]. The primary nonunion rate was highest using Kirschner wires, intermediate using tension band wires, and lowest using Compression screws; in a systematic review on this topic, this assumption could not be proven [12]. One possible explanation was that when combining the techniques into an analysis of compression versus non-compression, more advanced techniques such as tension bands were included—which show a higher complication rate than the compression screw—and therefore no evidence could be found. This might be why there are different studies which have shown a superiority of the compression screw over other implants [23,24]. In this biomechanical study, the compression forces of the different implants were not recorded—other studies in the future should further elaborate on this topic. However, there seems to be a tendency that techniques using compression—such as the cerclage, tension band or compression screw—provide higher primary stability and therefore more reliable results.

In this study, a fusion angle of 40° was chosen. Since there are fusion angles of 20–50° that are recommended depending on the finger, this was a compromise [25]. There are several publications on the effect of the chosen arthrodesis angle on the grip strength and function of the hand. Xu et al., was able to show that the resulting strength loss, although significant, did not differ between the range of 20–40° [26]. This is in accordance with the results of Dimitrova et. al., who were also able to show a significant decrease in grip strength in all fingers beside the index [27]. However, since the functional impact of a symptomatic osteoarthritic PIP joint is higher than that of arthrodesis, fusion is still a possible treatment option. Other biomechanical studies on the arthrodesis of the proximal interphalangeal joint have used fusion angles of 20° to 40°; therefore, the chosen 40° angle seemed a reasonable compromise [28,29,30]. The results of these studies are in accordance with the respective techniques of this study.

In previous biomechanical studies of the stability of the PIP joint arthrodesis by Millrose et al., as well as by Vonderlind et al., in 2019, formalin-fixed human cadaver specimens were used [28,29]. Cadaver specimens have long been used in biomechanical studies, but have different disadvantages compared to those of composite bone models. They are costly and, most importantly, may vary in their structure with respect to mineralization (osteoporosis) [31]. As the purpose of this study was to firstly compare the primary stability of different implants for PIP joint arthrodesis, composite bone was used, as a lot of specimens were necessary and confounders were to be excluded as much as possible.

The forces the implant materials in this biomechanical study were able to withstand exceed most of the activities of daily life [32,33]. This is also confirmed by the results of corresponding studies, which have shown that the forces acting on the middle finger joint during everyday activities such as typing on the computer, holding a pen or playing the piano using different grip shapes range from 1.9 to 19.40 N, or that the maximum force required for flexion of the middle joints of the long fingers is 8.79 N [15,34]. Therefore, a protective immobilization of the arthrodesis might not be necessary—except for the plates—as long as the patient is capable of understanding and maintaining an early, functional, unencumbered rehabilitation. As has been shown in a systematic review of early active motion protocols after flexor tendon injury, the TAM (total active movement) of the finger is mostly preserved, which benefits the whole function of the hand [35].

Our biomechanical study has certain limitations: First of all, we did not analyze the primary stability of different fusion techniques at different angles. Since it is normally recommended to adjust the fusion angle to the respective finger—from 20° for the index up to 50° for the little finger—there might be different implants that might stabilize a lower or higher angle better, respectively. We did not analyze the resistance to torquing forces, which might be interesting—but those forces are not usually relevant. Additionally, we did not analyze the compression force applied by the implant at fusion and during application of the bending force; this might become important for clinical studies as there is a hypothesis that a compressed fusion site will heal primarily and therefore provide more reliable results [23].

A strength of this study is that it is the first biomechanical analysis which compares all the modern standard fusion techniques in such a way as to compare the primary stability of the implant in a PIP joint arthrodesis. This can be the basis of further clinical studies with and without immobilization after PIP joint fusion to better preserve the global function of the hand. By using standardized composite bone models, confounders such as low mineralization or other influences on the bone quality such as neighboring tissue lesion or injuries were excluded.

## 5. Conclusions

Based on the results of this biomechanical study, cerclages, tension bands and compression screws might be able to provide enough stability to withstand the forces induced by unencumbered activities of daily living and may warrant further clinical studies. Arthrodesis of the PIP joint using plates should be limited to special indications and always requires immobilization for the whole consolidation period.

## Figures and Tables

**Table 1:** Extension of PIP joint arthrodesis with different techniques.

	K Wire	Cerclage	Compression Wire	Tension Band	Compression Screw	Fixation Plate	Locking Plate
	*n* = 10	*n* = 10	*n* = 10	*n* = 10	*n* = 10	*n* = 10	*n* = 10
**39.5°**	3.16 ± 0.87	5.28 ± 3.56	3.43 ± 0.67	4.18 ± 1.31	4.60 ± 1.88	2.31 ± 0.09	3.32 ± 0.58
**39°**	4.57 ± 1.52	8.46 ± 5.67	5.09 ± 1.70	5.74 ± 2.10	7.68 ± 3.58	2.58 ± 0.14	4.29 ± 0.76
**38.5°**	6.17 ± 2.01	11.68 ± 6.95	8.05 ± 3.98	7.18 ± 2.76	11.03 ± 6.20	2.82 ± 0.16	5.06 ± 1.00
**38°**	7.80 ± 2.64	15.03 ± 8.04	11.47 ± 6.56	8.52 ± 3.69	14.36 ± 8.80	3.03 ± 0.19	5.91 ± 1.05
**37.5°**	9.37 ± 3.21	18.65 ± 8.82	14.73 ± 8.21	10.28 ± 3.97	17.48 ± 9.19	3.27 ± 0.21	6.72 ± 1.06
**37°**	10.95 ± 3.72	22.21 ± 9.46	17.99 ± 9.75	11.95 ± 4.41	22.67 ± 13.84	3.47 ± 0.27	7.46 ± 1.04
**36.5°**	12.52 ± 4.21	25.56 ± 10.16	21.65 ± 11.23	13.49 ± 4.89	27.36 ± 17.44	3.68 ± 0.25	8.24 ± 1.12
**36°**	14.49 ± 4.75	28.80 ± 11.04	26.24 ± 12.48	15.18 ± 5.29	32.26 ± 20.00	3.87 ± 0.26	8.93 ± 1.16
**35.5°**	16.21 ± 5.13	31.74 ± 11.70	31.10 ± 13.45	16.78 ± 5.58	37.12 ± 20.27	4.06 ± 0.28	9.57 ± 1.24
**35°**	17.97 ± 5.46	34.63 ± 12.37	35.69 ± 14.42	18.41 ± 5.82	40.40 ± 17.00	4.21 ± 0.30	10.27 ± 1.19
**34.5°**	19.60 ± 5.79	36.99 ± 12.54	39.89 ± 15.54	19.87 ± 5.98	45.71 ± 18.63	4.34 ± 0.29	10.97 ± 1.10
**34°**	21.20 ± 6.13	39.38 ± 12.93	43.73 ± 16.72	21.18 ± 5.98	52.08 ± 17.90	4.47 ± 0.31	11.51 ± 1.06
**33.5°**	22.74 ± 6.36	40.21 ± 13.37	47.17 ± 17.78	22.54 ± 6.11	55.29 ± 19.49	4.61 ± 0.32	11.97 ± 1.00
**33°**	24.53 ± 6.29	42.93 ± 13.17	49.44 ± 18.26	23.76 ± 6.10	60.27 ± 20.60	4.72 ± 0.33	12.41 ± 0.92
**32.5°**	26.26 ± 6.20	45.11 ± 13.23	53.06 ± 17.92	24.91 ± 6.18	61.47 ± 18.24	4.84 ± 0.34	12.87 ± 0.83
**32°**	27.89 ± 5.87	47.10 ± 13.30	56.88 ± 16.71	25.92 ± 6.29	63.01 ± 16.00	4.97 ± 0.35	13.25 ± 0.79
**31.5°**	29.35 ± 5.80	49.01 ± 13.39	60.43 ± 16.28	26.71 ± 6.29	64.25 ± 14.49	5.06 ± 0.36	13.68 ± 0.67
**31°**	30.85 ± 5.77	50.78 ± 13.55	63.42 ± 16.28	27.80 ± 6.49	65.15 ± 13.45	5.15 ± 0.36	14.09 ± 0.58
**30.5°**	32.18 ± 5.81	52.51 ± 13.58	66.19 ± 16.54	28.70 ± 6.50	66.35 ± 12.68	5.24 ± 0.35	14.43 ± 0.52
**30°**	33.60 ± 5.43	54.11 ± 13.69	68.53 ± 16.85	29.62 ± 6.83	68.12 ± 11.07	5.33 ± 0.34	14.69 ± 0.50

Data is presented as mean ± standard deviation.

**Table 2:** Flexion of PIP joint arthrodesis with different techniques.

	K Wire	Cerclage	Compression Wire	Tension Band	Compression Screw	Fixation Plate	Locking Plate
	*n* = 10	*n* = 10	*n* = 10	*n* = 10	*n* = 10	*n* = 10	*n* = 10
**40.5°**	2.80 ± 0.27	3.39 ± 1.41	3.37 ± 0.71	3.38 ± 0.82	4.06 ± 0.97	3.37 ± 1.35	3.81 ± 0.59
**41°**	3.71 ± 0.21	4.53 ± 2.58	4.69 ± 1.71	4.37 ± 1.69	6.00 ± 1.82	4.45 ± 2.64	5.50 ± 1.06
**41.5°**	4.81 ± 0.96	6.27 ± 4.01	6.07 ± 2.74	5.84 ± 2.23	7.97 ± 3.17	7.09 ± 7.78	7.85 ± 2.49
**42°**	5.90 ± 1.57	8.43 ± 5.53	7.53 ± 3.69	7.35 ± 2.87	10.01 ± 4.25	10.15 ± 13.59	10.60 ± 4.75
**42.5°**	6.94 ± 1.93	10.84 ± 7.13	9.00 ± 4.67	8.91 ± 3.58	11.94 ± 5.15	13.34 ± 19.38	12.71 ± 4.07
**43°**	8.08 ± 2.36	13.28 ± 8.85	10.39 ± 5.52	10.46 ± 4.31	13.94 ± 6.01	16.73 ± 25.39	16.16 ± 5.69
**43.5°**	9.29 ± 2.73	15.76 ± 10.48	11.66 ± 6.20	11.95 ± 4.97	15.88 ± 6.49	20.71 ± 31.61	21.46 ± 10.50
**44°**	10.60 ± 3.15	18.22 ± 12.08	12.90 ± 6.80	13.28 ± 5.65	17.58 ± 6.65	24.48 ± 37.50	26.77 ± 14.80
**44.5°**	11.83 ± 3.59	20.86 ± 13.36	14.19 ± 7.32	14.88 ± 6.35	19.06 ± 6.65	28.06 ± 43.89	31.88 ± 18.50
**45°**	12.92 ± 3.91	23.54 ± 14.46	15.67 ± 7.85	16.67 ± 7.10	20.10 ± 6.19	22.19 ± 19.86	36.67 ± 21.96
**45.5°**	13.94 ± 4.20	26.07 ± 15.49	17.14 ± 8.47	18.29 ± 7.86	21.18 ± 5.95	27.42 ± 29.08	41.10 ± 25.05
**46°**	14.97 ± 4.44	28.48 ± 16.37	18.58 ± 8.99	19.97 ± 8.54	22.37 ± 5.99	33.00 ± 36.73	45.39 ± 28.24
**46.5°**	16.29 ± 4.54	30.72 ± 17.18	20.17 ± 9.10	21.67 ± 9.37	23.62 ± 6.18	39.51 ± 44.44	48.50 ± 30.61
**47°**	17.51 ± 4.79	32.88 ± 17.91	21.91 ± 9.64	23.37 ± 10.26	25.01 ± 6.45	47.12 ± 50.70	56.21 ± 34.41
**47.5°**	18.55 ± 5.09	34.97 ± 18.66	23.46 ± 10.27	25.21 ± 11.33	26.58 ± 6.68	52.43 ± 56.39	65.25 ± 39.34
**48°**	19.55 ± 5.27	36.96 ± 19.37	24.91 ± 10.92	27.12 ± 12.27	28.23 ± 6.93	61.08 ± 59.67	74.42 ± 44.06
**48.5°**	20.75 ± 5.17	38.85 ± 20.00	26.14 ± 11.39	29.27 ± 12.95	29.82 ± 7.24	73.03 ± 59.49	84.60 ± 46.22
**49°**	22.14 ± 5.21	40.76 ± 20.47	27.31 ± 11.72	31.45 ± 13.83	31.41 ± 7.56	88.06 ± 58.09	100.47 ± 49.59
**49.5°**	23.36 ± 5.41	42.49 ± 20.96	28.40 ± 11.97	33.48 ± 14.72	33.03 ± 7.76	103.22 ± 56.54	100.71 ± 51.63
**50°**	24.41 ± 5.61	44.00 ± 21.22	29.57 ± 12.17	35.41 ± 15.56	34.68 ± 7.97	118.37 ± 55.07	118.51 ± 56.84

Data is presented as mean ± standard deviation.

**Table 3:** Differences in extension between the methods for different angles.

	37.5°	35.0°	32.5°	30.0°
Compression screw [N]	17.5; *p* = 0.006	40.4; *p* = 0.002	61.5; *p* = 0.001	68.1; *p* < 0.001
Compression wire [N]	14.7; *p* = 0.142	35.7; *p* = 0.016	53.1; *p* = 0.004	68.5; *p* < 0.001
Cerclage [N]	18.6; *p* = 0.016	34.6; *p* = 0.009	45.1; *p* = 0.006	54.1; *p* = 0.003
Tension band [N]	10.3; *p* = 0.510	18.4; *p* = 0.844	24.9; *p* = 0.617	29.6; *p* = 0.145
K wire [N]	9.4	18.0	26.3	33.6
Locking plate [N]	6.7; *p* = 0.044	10.3; *p* = 0.003	12.9; *p* < 0.001	14.7; *p* < 0.001
Fixation plate [N]	3.3; *p* < 0.001	4.2; *p* < 0.001	4.8; *p* < 0.001	5.3; *p* < 0.001

Results are reported as mean value for each method and angle; *p*-values are derived from a pairwise t-test in comparison to the K wire method as reference; green = statistically significantly more stable than K wire, red = statistically significantly less stable than K wire.

**Table 4:** Differences in flexion between the methods for different angles.

	42.5°	45.0°	47.5°	50.0°
Locking plate [N]	12.7; *p* = 0.004	36.7; *p* = 0.011	65.2; *p* = 0.006	118.5; *p* < 0.001
Fixation plate [N]	13.3; *p* = 0.310	22.2; *p* = 0.147	52.4; *p* = 0.081	118.4; *p* < 0.001
Cerclage [N]	10.8; *p* = 0.153	23.5; *p* = 0.062	35.0; *p* = 0.030	44.0; *p* = 0.030
Tension band [N]	8.9; *p* = 0.217	16.7; *p* = 0.260	25.2; *p* = 0.213	35.4; *p* = 0.125
Compression screw [N]	11.9; *p* = 0.031	20.1; *p* = 0.012	26.6; *p* = 0.004	34.7; *p* < 0.001
Compression wire [N]	9.0; *p* = 0.263	15.7; *p* = 0.397	23.5; *p* = 0.232	29.6; *p* = 0.212
K wire [N]	6.9	12.9	18.5	24.4

Results are reported as mean value for each method and angle; *p*-values are derived from a pairwise t-test in comparison to the K wire method as reference; green = statistically significantly more stable than K wire, red = statistically significantly less stable than K wire.

## Data Availability

The data presented in this study are available on request from the corresponding author.
